# Comparative Genome Analyses of *Serratia marcescens* FS14 Reveals Its High Antagonistic Potential

**DOI:** 10.1371/journal.pone.0123061

**Published:** 2015-04-09

**Authors:** Pengpeng Li, Amy H. Y. Kwok, Jingwei Jiang, Tingting Ran, Dongqing Xu, Weiwu Wang, Frederick C. Leung

**Affiliations:** 1 Bioinformatics Center, Nanjing Agricultural University, Nanjing, China; 2 College of Life Sciences, Nanjing Agricultural University, Nanjing, China; 3 School of Biological Sciences, University of Hong Kong, Hong Kong Special Administration Region, China; Graz University of Technology (TU Graz), AUSTRIA

## Abstract

*S*. *marcescens *FS14 was isolated from an *Atractylodes macrocephala Koidz *plant that was infected by *Fusarium oxysporum* and showed symptoms of root rot. With the completion of the genome sequence of FS14, the first comprehensive comparative-genomic analysis of the *Serratia* genus was performed. Pan-genome and COG analyses showed that the majority of the conserved core genes are involved in basic cellular functions, while genomic factors such as prophages contribute considerably to genome diversity. Additionally, a Type I restriction-modification system, a Type III secretion system and tellurium resistance genes are found in only some *Serratia* species. Comparative analysis further identified that *S*. *marcescens* FS14 possesses multiple mechanisms for antagonism against other microorganisms, including the production of prodigiosin, bacteriocins, and multi-antibiotic resistant determinants as well as chitinases. The presence of two evolutionarily distinct Type VI secretion systems (T6SSs) in FS14 may provide further competitive advantages for FS14 against other microbes. To our knowledge, this is the first report of comparative analysis on T6SSs in the genus, which identifies four types of T6SSs in *Serratia* spp.. Competition bioassays of FS14 against the vital plant pathogenic bacterium *Ralstonia solanacearum* and fungi *Fusarium oxysporum *and *Sclerotinia sclerotiorum* were performed to support our genomic analyses, in which FS14 demonstrated high antagonistic activities against both bacterial and fungal phytopathogens.

## Introduction

The bacterial strain FS14 was isolated from an *Atractylodes macrocephala Koidz* plant infected by the pathogen *Fusarium oxysporum*, which is a causative agent of the root-rot disease [1]. The isolated red-pigmented bacteria were found to secret thermostable DNase and protease in our previous study [1]. With the combined use of morphological, biochemical and genetic characterization via 16S rDNA sequencing, the isolate was classified into the genus *Serratia* and designated as strain FS14. As the strain was found along with phytopathogens in diseased plants, we hypothesized that it has antagonistic potential against the pathogens.

*Serratia* is a genus of Gram-negative, facultatively anaerobic, rod-shaped bacteria of the *Enterobacteriaceae* family. They are ubiquitous organisms with diverse habitats including water, soil, plants, small mammals, and humans. The typical species of the genus is *Serratia marcescens*, well known as an opportunistic human pathogen involved in hospital acquired infections [2]. *Serratia* species with similar sites of isolation to strain FS14 include the endophytic *S*. *proteamaculans* found to promote plant growth via the production of specific compounds such as lipo-chitin oligosaccharides [3] and *S*. *plymuthica* which is considered a plant growth promoting rhizobacteria [4] and an antagonistic bacteria against phytopathogens since they produce a wide range of antimicrobial compounds [5].

Antagonistic bacteria exploit diverse strategies to outperform their competitors. The most characterized antimicrobial compound in *Serratia* is the red pigmented prodiginine, of which five types have been identified so far (prodigiosin, undecylprodigiosin, cycloprodigiosin, cyclononylprodigiosin, and butyl-meta-cyclo-heptylprodiginine) [6]. Prodigiosin is commonly produced by environmental isolates of *S*. *marcescens*, but not the clinical isolates [7]. In addition to its antibacterial, antifungal and antiprotozoal properties, prodiginine was recently reported to exhibit immunosuppressive and anticancer traits [8].

Another central strategy employed is manifested via the protein secretion systems, through which Gram-negative bacteria secrete effectors to their exterior [9]. In particular, the Type VI secretion system (T6SS), the most recently described of the six, has highly versatile functions, which include eukaryotic and bacterial cell targeting, gene regulation, conjugation and cellular adhesion [10]. Recent studies demonstrated that T6SSs in *S*. *marcescens* and other bacteria, *e*.*g*. *Vibrio cholerae*, *Burkholderia thailandensis* [11, 12], can target other bacterial competitors resulting in either growth inhibition or death.

Compared with *Escherichia* and *Salmonella*, which are also members of the *Enterobacteriaceae* family, there are relatively fewer—only 13—genomes of *Serratia* sequenced and reported to NCBI (ftp://ftp.ncbi.nlm.nih.gov/genomes/Bacteria/): *S*. *marcescens* WW4 isolated from a paper machine [13], *S*. *marcescens* Db11 pathogen of drosophila, *S*. *marcescens* FGI 94 associated with leaf-cutter ant fungus garden [14], *S*. *proteamaculans* 568 with a detailed genome analysis on chitinase production [3], *S*. *liquefaciens* ATCC27592, *S*. *fonticola* RB-25 isolated from a waste landfill [15], three plant associated *S*. *plymuthica* strains AS13 [16], AS12, AS9, S13 [17] and 4Rx13 that exhibit plant-growth-promoting activities and *Serratia symbiotica Cinara cedri uid82363* which coexists with *B*. *aphidicola* in aphid.

In this study, we present the first comprehensive comparative-genomic analysis of the genus *Serratia*, which helps to identify the antimicrobial compounds they secrete and other antagonistic genomic elements they harbor, *e*.*g*. Type VI secretion system, antibiotic-resistant genes and chitinases. In addition, we explore the antagonistic potential of strain FS14 against common fungal and bacterial phytopathogens.

## Materials and Methods

### Isolation and Total DNA extraction of *Serratia marcescens* FS14

*Serratia marcescens* FS14 was isolated in May 2009 from an *Atractylodes macrocephala Koidz* plant infected by *Fusarium oxysporum* in Jiangsu Province, China. A single isolate was grown in LB media and incubated at 28°C until they reached the exponential growth phase. The bacteria were then harvested by centrifugation and their genomic DNA was extracted according to the JGI bacterial DNA isolation CTAB protocol (http://my.jgi.doe.gov/general/protocols/JGI-Bacterial-DNA-isolation-CTAB-Protocol-2012.pdf).

### Antibiotic-sensitivity tests

Antibiotic-sensitivity tests were performed by spreading bacterial suspensions on culture plates [18] and applying discs impregnated with different antibiotics (Hangzhoutianhe, China) including gentamycin (10 μg), penicillin G (10 IU), tetracycline (30 μg), rifampicin (5 μg), novobiocin (30 μg), neomycin (30 μg), spectinomycin (100 μg), ampicillin (10 μg), amoxicillin (10 μg), acetylspiramycin (30 μg), chloramphenicol (30 μg), erythromycin (15 μg), kanamycin (30 μg) and polymyxin B sulfate (300 μg). For all the above tests, each plate was incubated at 28°C for 24 hours under aerobic condition and the zones of inhibition were interpreted according to the manufacturer's instructions. All assays were performed in triplicate.

### Pyrosequencing and complete genome assembly of *S*. *marcescens* FS14

To confirm the purity of *S*. *marcescens* FS14 genomic DNA, the 16S rDNA-specific region was amplified and 20 individual positive clones were sequenced by Genetic Analyzer 3130 (Invitrogen, Grand Island, US). BLASTn analysis [19] revealed that the *S*. *marcescens* FS14 16S rDNA sequence has a high similarity to that of species from the genus of *Serratia* available on GenBank. The quality of genomic DNA was evaluated by 0.8% agarose gel electrophoresis and Nanodrop2000 (Thermo Scientific, Waltham, US), and the quantity was evaluated by the Quant-iT Picogreen dsDNA kit (Invitrogen).

A whole genome shotgun library was generated with 500ng of *S*. *marcescens* FS14 genomic DNA. The shotgun sequencing procedure was performed using a Roche GS Junior Rapid Library Preparation Kit, per the manufacturer’s instructions (Roche, Basel, Switzerland). In addition, an 8kb span paired end library was generated with 15μg of *S*. *marcescens* FS14 genomic DNA. The paired end sequencing procedure was performed using a Roche GS Junior Paired End Library Preparation Kit, per the manufacturer’s instructions (Roche). One shotgun run and two paired end runs were performed on individual libraries prepared with the same genomic DNA sample. After sequencing, the raw data was assembled by Newbler 2.7 (Roche) with default parameters using paired end reads as an orientation guide. Primer pairs were designed along sequences flanking the gap regions and PCR gap-filling was followed by Sanger sequencing. The complete genome of *S*. *marcescens* FS14 was submitted to NCBI, GenBank accession number is CP005927, and it is publicly available for download at http://www.ncbi.nlm.nih.gov/nuccore/CP005927.

### Genome annotation

Genome annotation and analysis of *S*. *marcescens* FS14 was performed using NCBI Prokaryotic Genomes Automatic Annotation Pipeline (PGAAP) [20]. BLASTp [19] was applied to align the amino acid sequences against the COG database [21], VFDB database [22], and ARDB database [23]. Amino acid sequences with an alignment length over 90% of its own length and over 40% match identity (sequences annotated against COG database were filtered using over 20% match identity) were chosen and the descriptions of the best hits (with the highest alignment length percentage and match identity) were assigned as annotations of the predicted genes. All predicted amino acid sequences of *S*. *marcescens* FS14 were submitted to the KEGG database [24] for automatic pathway annotation (http://www.genome.jp/kaas-bin/kaas_main). All annotated pathways were manually downloaded and curated by in-house Perl scripts. PHAST (PHAge Search Tool)[25], available at http://phast.wishartlab.com/ was used for prophage identification. Genomic islands were predicted through IslandViewer [26] using the IslandPath-DIMOB method [27].

### Phylogenetic and phylogenomic analysis

For phylogenomic analysis, 11 complete genomic sequences of *Serratia* spp. (*S*. *marcescens* WW4 [CP003959], *S*. *marcescens* Db11 [HG326223], *S*. *marcescens* FGI 94 [CP003942], *S*. *proteamaculans* 568 [CP000826], *S*. *plymuthica AS13* [CP002775], *S*. *plymuthica* AS9 [NC_015567], *S*. *plymuthica* AS12 [NC_015566], *S*. *plymuthica* S13 [CP006566], *S*. *plymuthica* 4Rx13 [CP006250], *S*. *liquefaciens* ATCC27592 [CP006252], and *S*. *fonticola* RB-25 [CP007044]) were downloaded from NCBI (ftp://ftp.ncbi.nlm.nih.gov/genomes/Bacteria/). The genomic sequence of *Serratia symbiotica Cinara cedri* uid82363 has a genome size of only 1.8 Mb due to its endosymbiotic nature [28]; therefore, it was not included in the analysis. The orthologous genes were identified using BLAT [29] to match and align *S*. *marcescens* FS14 (CP005927) CDSs to all annotated genes from other *Serratia* spp. genomes. BLAT hits that were presented as single copies in *Serratia* spp. and cover over 90% of alignment length of FS14 genes were considered core genes. All of the core genes were aligned by MUSCLE [30] and randomly concatenated together. Finally, the phylogenomic tree was generated using these concatenated aligned genes with the GTR+G+I substitution model by MrBayes [31]. The chain length was set to 10,000,000 (1 sample/1000 generations). The first 2,000 samples were discarded as burn in after scrutinizing the trace files of two independent runs with Tracer v1.4 (http://tree.bio.ed.ac.uk/software/tracer/).

Since *S*. *plymuthica* AS9, AS12 and AS13 share a very high similarity, AS13 was chosen as the representative for all the following comparative analyses.

Pan genome analysis was performed using reciprocal BLAT between the ten *Serratia* spp. genomes, and the number of orthologs shared between them was calculated by in-house Perl scripts. Genomes of *S*. *marcescens* were aligned by Mauve [32] with Progressive Mauve [33] based on default parameters for investigation of potential genomic rearrangements.

The molecular phylogenetic tree was constructed using the maximum likelihood (ML) method (MEGA5) [34] based on the Poisson correction model [35] with concatenated amino acid sequences of two proteins, GyrB and RpoD, which are conserved in bacteria. They were retrieved from 16 different strains of *Serratia* and an *E*. *coli* K-12 publicly accessible on GenBank. A bootstrap consensus tree inferred from 100 replicates was chosen. The multiple alignments of protein sequences were built by CLUSTALW [36]. The same procedures and models were applied to a dataset consisting of 8 concatenated proteins—PigA(RedW), PigC(RedH), PigF(RedI), PigG(RedO), PigH(RedN), PigI(RedM), PigK(RedY) and PigM(RedV), all of which are homologues among different prodiginine-producing bacteria. Phylogenetic analysis of the Type III secretion system (T3SS) was performed based on a previous study [37]. Representatives of bacteria with T3SS were chosen to perform the phylogenetic analysis of T3SSs in *Serratia*. The multiple alignments of the T3SS ATPase sequences were built by CLUSTALW [36], and then a ML tree was constructed based on the Poisson correction model [35] with a bootstrap value of 100 by MEGA5 [34]. The T6SSs of *Serratia* were manually identified and the synteny study of the T6SS clusters was performed based on SyntTax [38] using default parameters and followed by manual confirmation.

### Bacterial and fungal antagonistic bioassays

All biological samples involved in the bioassays designed to investigate the antibacterial and antifungal activities of *S*. *marcescens* FS14 were collected in Jiangsu province, China. The bacterial isolate, *Ralstonia solanacearum* NJ (RSNJ), was isolated from a tomato plant. The phytopathogenic fungus *Fusarium oxysporum* was isolated from the same plant that *S*. *marcescens* FS14 was isolated from, and *Sclerotinia sclerotiorum* was isolated from an infected *Brassica napus* ZY5. For fungal antagonistic assays, each fungus was grown for four days at 28°C and a 0.5cm-diameter agar with mycelia was cut and placed at a spot 2 cm away from the edge of the PDA plate. The overnight culture of FS14 was streaked across the middle of the plate [39]. Controls were set up without FS14. Fungal growth was observed for one week. All assays were performed in triplicate.

To prepare for the antibacterial competition assay, bacterial cultures were grown overnight in improved nutrient broth (1% glucose, 0.5% tryptone, 0.05% yeast extract and 0.3% beef extract) and adjusted to an OD_600_ of 0.5. For the biofilm culture, FS14 and RSNJ were mixed at a ratio of 1:1. 25μl of this mixture was spotted onto agar plates and incubated at 28°C. After 8-hour incubation, biofilm samples were recovered from the spot for quantification using sterile metal loops and then re-suspended in 1 ml improved nutrient broth. The recovery of viable cells is reported as the total number of recovered cells per biofilm co-culture spot. The control mixture was set up without FS14 and contained a 1:1 ratio of sterile nutrient broth to RSNJ. For the planktonic culture, FS14 and *R*. *solanacearum* NJ cells were adjusted to an OD_600_ of 0.1 and equal volumes (15 ml) of FS14 and RSNJ were mixed at a ratio of 1:1 in a 150 ml flask and cultured in a shaking incubator (200rpm at 28°C). One milliliter of culture was collected for quantification at 2, 4, 6, 8, 10, 12 and 24 hours after incubation. Then the quantities of FS14 and RSNJ were measured by serial dilutions and presented as Log CFU (colony-forming unit)/ml. Serial diluted samples were plated out on improved nutrient agar (w/v 1.5%) plates and CFU was counted after an incubation of 48 hours at 28°C. FS14 and RSNJ can be distinguished by color and antibiotic selection. Samples containing only FS14 or RSNJ were set up as controls. Cell number of each bacterium spotted onto the biofilm coculture plate at t = 0 hour is ~1 x 10^6^, and concentration of each bacterium in the planktonic coculture at t = 0 hour is ~1.2 x 10^6^ CFU/ml. Three independent experiments were performed for each assay.

## Results and Discussion

### Complete genome sequencing

The *Serratia* sp. FS14 genome is composed of a single circular chromosome of ~5.25 Mbp with a genomic GC content of 59.46% (S1 Fig) and a total of 4761 predicted coding sequences (CDSs). 91 tRNA genes and 6 rRNA operons were found in the FS14 genome. Genome statistics of the ten *Serratia* strains with complete genomes are shown in Table 1. FS14 has a similar genome size with *S*. *marcescens* WW4 and Db11. *S*. *marcescens* FGI 94 has a much smaller genome (~4.86 Mbp) with 4361 CDSs. CDSs that are absent in FGI94 are mainly transcriptional regulators, ATP-dependent transporters, chitinases and fimbrial biosynthetic proteins, all of which are not essential for bacterial survival [14, 40, 41, 42] and may not be necessary in its symbiotic lifestyle with the fungus; for instance, chitinases may antagonize its symbiotic fungus partner. This is partly in accordance to previous hypotheses that symbiotic bacteria genomes usually experience a reductive process and retained only the most essential functions [43, 44]. The genomic GC content of the analyzed *Serratia* strains ranges from 50.9% to 59.6%. The four *S*. *marcescens* strains have the highest GC content (around 59%) and *S*. *fonticola* RB-25 has the lowest GC content of 50.9%. No plasmid was identified in FS14, while *S*. *marcescens* WW4, *S*. *proteamaculans* 568, *S*. *plymuthica* 4Rx13 and *S*. *liquefaciens* ATCC27592 contained one plasmid each. No similarity can be observed between the plasmids in different strains of *Serratia*, which contributes to the variation of genomic content.

**Table 1:** Genome Statistics of *S*. *marcescens* FS14 and other *Serratia* spp..

	*S*. *marcescens*				*S*. *proteamaculans*	*S*. *plymuthica*			*S*.*liquefaciens*	*S*. *fonticola*
	FS14	WW4	Db11	FGI 94	568	AS13	S13	Rx13	ATCC 27592	RB-25
Genome size (Mb)	5.24	5.24	5.11	4.86	5.5	5.44	5.47	5.4	5.28	5.49
GC content %	59.5	59.6	59.5	58.9	55	56	56.2	56.2	55.5	50.9
No. of chromosome	1	1	1	1	1	1	1	1	1	1
No. of plasmid	0	1	0	0	1	0	0	1	1	0
Predicted CDSs	4761	4809	4709	4361	4942	4951	4991	4695	4894	4825
No. of tRNAs	91	79	88	83	85	87	85	81	79	77
No. of rRNA operons	6	7	7	7	7	7	7	7	6	7
Percentage of ARDB annotated CDS %	0.46	0.42	0.38	0.6	0.49	0.42	0.45	0.45	0.45	0.33
Site of isolation	plant	paper machine	drosophila	Fungus garden	plant	plant	plant	soil	milk	landfill

### Taxonomic classification and comparative analysis of the genome

**Fig 1:**
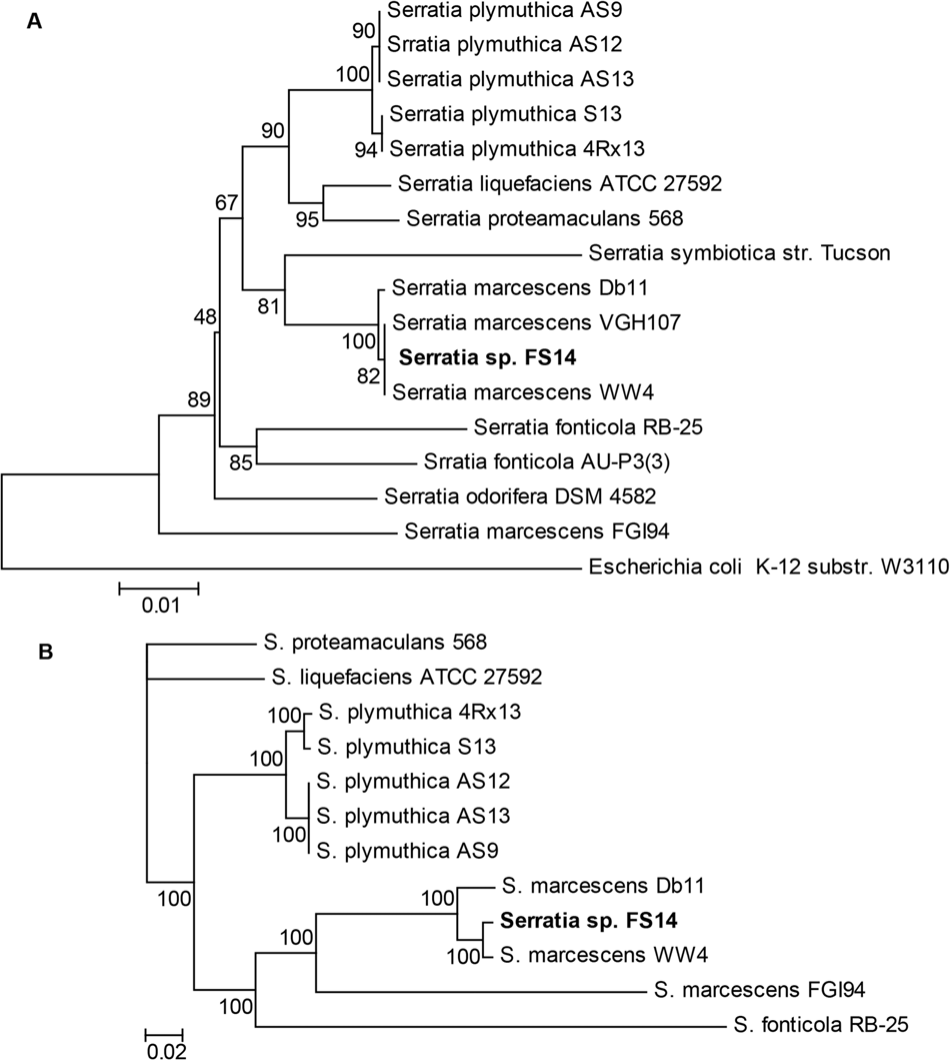
Taxonomic classification of *Serratia marcescens* FS14. (A) Maximum Likelihood Tree using Poisson correction model [35] and with 100 bootstrap replicates was constructed based on 2 conserved proteins among all bacteria—GyrB and RpoD—by MEGA 5 [34]. (B) The phylogenomic tree was constructed by MrBayes [17] using the random concatenation of 731 aligned core genes as the dataset and GTR + G+ I as the substitution model. The chain length was set to 10,000,000 (1 sample/1000 generations) whilst the burn-in was set as 2000. Posterior probabilities are denoted at nodes.

The bacterial isolate FS14 was classified into the genus *Serratia* according to its morphology, physiology, biochemistry, and 16S rDNA sequence [45]. In this study, taxonomic classification of strain FS14 was refined by two *in silico* analyses. In the phylogenetic tree constructed based on the two proteins conserved among bacteria—GyrB and RpoD [46, 47], *Serratia* sp. FS14 was found to form a close cluster with *S*. *marcescens* VGH107 (a clinical isolate), *S*. *marcescens* WW4 and *S*. *marcescens* Db11 with a high bootstrap value (100) distinct from clusters formed by other *Serratia* spp. (Fig 1A). Interestingly, *S*. *marcescens* FGI94 did not form a close cluster with the other strains of the same species.

To better elucidate the taxonomic classification of FS14, a phylogenomic tree was constructed based on the 731 core genes (~19% of the FS14 genome) identified from the genome of FS14 and the other nine complete *Serratia* genomes publicly available. *Serratia* sp. FS14 was shown to cluster closely with *S*. *marcescens* WW4 with a high posterior probability (100) (Fig 1B), thus strongly advocating the notion that strain FS14 belongs to the species *S*. *marcescens*. Though FS14 was isolated from plants, it was unexpectedly found to be of a closer phylogenomic origin to the paper machine-isolated *S*. *marcescens* WW4 and the drosophila pathogen *S*. *marcescens* Db11 rather than the plant-associated *S*. *plymuthica* and *S*. *proteamaculans* [16, 48].

Mauve [32] analysis showed that the synteny between *S*. *marcescens* is not very conserved (S2 Fig). Chromosomal reorganizations are commonly observed along the whole stretch of the four genomes. In particular, a large region of ~3600–3700 kb in FS14 cannot be found in FGI 94, and further analysis identified these genes mainly as genomic factors associated with antagonistic traits of bacteria, such as the prodigiosin biosynthesis cluster, bacteriocins, iron uptake systems, chitinases and protein secretion systems, which, as previously mentioned, might not be necessary or even beneficial to its symbiotic lifestyle with fungus.

**Fig 2:**
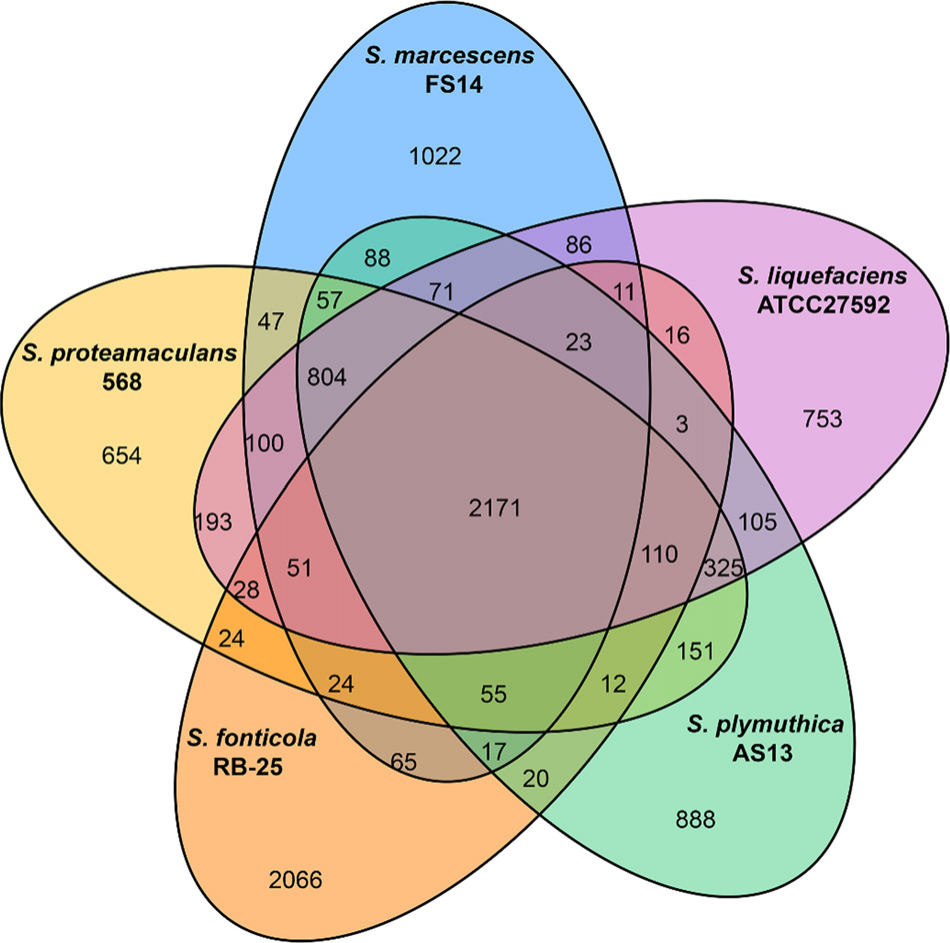
Venn diagram of five representative *Serratia* spp.. Five representative genomes, *S*. *marcescens* FS14 (CP005927), *S*. *proteamaculans* 568 (CP000826), *S*. *plymuthica* AS13 (CP002775), *S*. *liquefaciens* ATCC27592 (CP006252), and *S*. *fonticola* RB-25 (CP007044) were selected to illustrate the Venn diagram. The Venn diagram was not drawn in proportion; it sole purpose is to illustrate the common CDSs shared between the five strains. The overlapping regions represent CDSs shared with respective strains. The number outside the overlapping regions indicates the number of CDSs in each genome without homologs in other *Serratia* genomes.

The pan-genome analysis demonstrated that the ten *Serratia* strains shared 1964 (~41% of the predicted CDSs in FS14) genes in their genomes and most of them were assigned with general cellular functions (S1 Table). As shown in the Venn diagram of the five representative *Serratia* genomes (Fig 2), they share 2171 CDSs, corresponding to 44% to 46% of all CDSs in these genomes. The unique CDS percentages are 13%, 15%, 18% and 21% for *S*. *proteamaculans* 568, *S*. *liquefaciens* ATCC27592, *S*. *plymuthica* AS13 and *S*. *marcescens* FS14, respectively. *S*. *fonticola* RB-25 has the highest number (~43%) of unique genes including a tellurium resistance gene cluster (Z042_12505–12590). A type I restriction-modification (RM) system, unique among the *Serratia* spp., was identified on a putative genomic island GI01 of FS14 (S1 Fig). This putative Type I RM system may protect specific DNA restriction sites from endonucleases and play a role in the regulation of gene expression, as previously reported for a similar Type I RM system (sharing ~72% to 93% identities) in *Yersinia pseudotuberulosis* [49]. Interestingly, *S*. *marcescens* FGI 94 was revealed to encode a larger percentage (5.41% of its total CDSs) of genes potentially contributing to virulence than the insect pathogen *S*. *marcescens* Db11 (4.93%) [50]. In addition, FGI 94 encodes a unique Type III secretion system (T3SS) gene cluster that has a much higher GC content (66.12%) than the rest of its genome (58.9%), implicating this cluster may have been acquired through horizontal gene transfer. Phylogenetic analysis supports that it may originate from bacteria of other genera (S3 Fig); however, caution must be taken as multiple gene loss events in other *Serratia* spp. are improbable, though not impossible. In general, most of the unique genes in each strain are hypothetical proteins or are associated with prophage regions. 33 putative prophage regions are identified across the ten *Serratia* genomes, and FS14 encodes a unique ~53kb prophage region of *Enterobacteria* phage BP-4795 on its genomic island GI02 (S1 Fig, S2 Table).

Orthologs are genes in different species that have evolved from a common ancestral gene via speciation and often retain the same function in the course of evolution. Comparing orthologs is important to identify events of gene gain or loss. Clusters of orthologous groups (COGs) [51] provide genome-scale analysis of protein function prediction. Major COG-annotated class distribution is conserved between *Serratia* strains (S3 Table, S4 Fig). As expected, in all analyzed *Serratia* strains, the majority of COG-annotated genes are involved in basic cellular functions such as amino acid transport and metabolism (COG class [E]), transcription ([K]) and carbohydrate transport and metabolism ([G]); up to ~ 12% of the annotated genes have unknown ([S]) or predicted functions ([R])in the COG database.

### Antagonistic effect on phytopathogens of *S*. *marcescens* FS14

Bioassays were designed to investigate the antagonistic activities of FS14 against plant bacterial and fungal pathogens. *Fusarium oxysporum*, *Sclerotinia sclerotiorum* and *Ralstonia solanacearum* were used as the indicator microorganisms in antagonistic assays following the methods in previous reports [52, 53].

**Fig 3:**
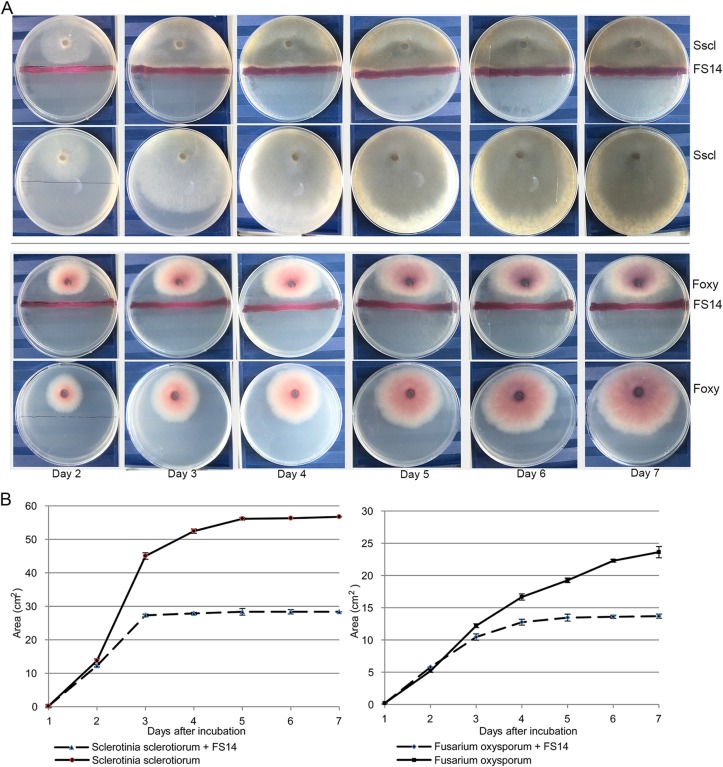
Antagonistic activities of *Serratia marcescens* FS14 against fungi. (A) Visualization of the FS14- *Sclerotinia sclerotiorum* (Sscl) and FS14-*Fusarium oxysporum* (Foxy) confrontation assays, 2–7 days after inoculation of Sscl (1^st^ and 2^nd^ rows) and Foxy (3^rd^ and 4^th^ rows) in the presence of *S*. *marcescens* FS14 (1^st^ and 3^rd^ rows) or in its absence (2^nd^ or 4^th^ rows); (B) Growth of *F*. *oxysporum* and *S*. *sclerotiorum* with and without FS14 were closely monitored. Challenging fungi were grown on PDA plates as described in Materials and Methods, the areas of growth of hyphae (in cm^2^) was measured. Numbers show an average of 3 plates, and error bars show standard errors of the means.

In the bacterial-fungal confrontation assays, FS14 was co-inoculated with *F*. *oxysporum* and *S*. *sclerotiorum*, both of which are common plant pathogens. As shown in Fig 3, compared to the control without FS14, the mycelia of both fungi were clearly inhibited by the FS14 colony streaked across the agar plates as early as the second day of inoculation. The fungal mycelia also turned a darker color at the later stages of bacteria-fungi interaction. Both fungi did not grow past the FS14 colony, though the non-contact inhibition seemed to be more evident in *F*. *oxysporum* than *Sclerotinia sclerotiorum*. These results indicate that FS14 can significantly suppress the growth of phytopathogenic fungi through non-contact inhibition, an ability which may be attributed to extracellular secretion of antifungal substances such as chitinases.

**Fig 4:**
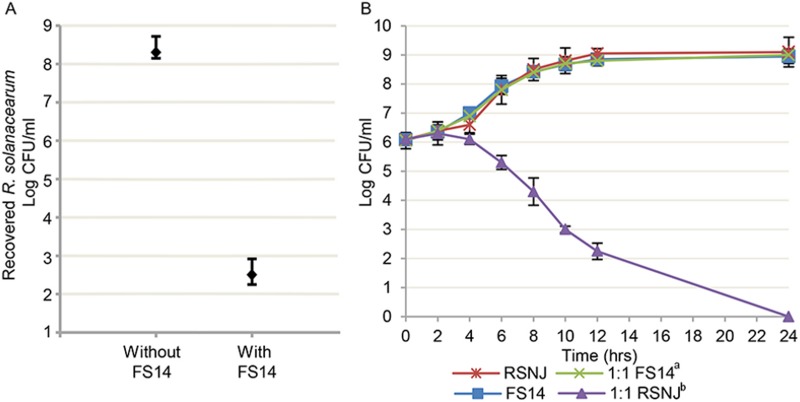
Antagonistic activity of *Serratia marcescens* FS14 against *Ralstonia solanacearum* NJ. (A) Recovery of viable *R*. *solanacearum* (RSNJ) cells after 8 hours of co-culture with (initial ratio of 1:1) and without FS14 at 28°C (see Materials and Methods for details); (B) The planktonic culture with quantities of *S*. *marcescens* FS14 (FS14) and *R*. *solanacearum* NJ (RSNJ) recorded at 0, 2, 4, 6, 8, 10, 12 and 24 hours after incubation. Numbers show an average of three replications, and error bars show standard errors of the means. a: the quantities of FS14 in the samples collected from the planktonic culture containing 1:1 of FS14 and RSNJ. b: the quantities of RSNJ in the samples collected from the planktonic culture containing 1:1 of FS14 and RSNJ.

In the bacteria-bacteria competition assay, *S*. *marcescens* FS14 was co-cultured with a target bacterium *R*. *solanacearum*, a devastating phytopathogenic bacterium which causes wilt in a wide range of host plants [54]. In the biofilm culture, when *R*. *solanacearum* NJ (RSNJ) was co-cultured with FS14 at an initial cell ratio of 1:1 for an 8-hour incubation, the final ratio of recovered cells from the RSNJ and FS14 co-culture was ~1:10^4^ (RSNJ: FS14). In comparison, a 10^6^ times increase in the number of viable RSNJ cells was recovered when they were cultured with medium only after the same period of incubation (Fig 4A). A similar result was observed when FS14 and RSNJ were co-cultured at an initial ratio of 1:1 in the planktonic culture (Fig 4B). These results imply that *S*. *marcescens* FS14 also possesses an antagonistic potential against the bacterial phytopathogen.

In the comparative genome analysis of FS14, genomic elements which may be involved in its antagonistic activities against fungi or bacteria are identified. These include antibiotic-resistant genes, Type VI secretion systems, prodigiosin and other antimicrobial compounds, and chitinases.

### Antibiotic-resistant traits

Many bacteria have evolved detoxification determinants or antibiotic resistance to counter antibiotics in competition against other microbes. Genomic analysis of *S*. *marcescens* FS14 showed that 22 (0.46%) out of 4761 predicted CDSs can be identified in the ARDB database (Table 2), suggesting it is a multi-drug resistant strain. The drosophila pathogen *S*. *marcescens* Db11 exhibits the highest number of antibiotic resistant genes (~0.6%, Table 1). Comparative study of FS14 and 9 other *Serratia* showed that all of them have potential resistance against four kinds of antibiotics: bacitracin, cephalosporin, fosmidomycin and penicillin. Interestingly, in the antibiotic sensitivity tests, FS14 exhibited resistance to vancomycin (Table 3), but the genomic analysis found no potential vancomycin-resistant genes in the FS14 genome, which may suggest that FS14 has either developed novel operons to resist vancomycin, or the antibiotic-resistant gene database is not comprehensive, or the presence of a multi-functional drug efflux pump is at work in the bacteria. Despite minor discrepancies, the experimental results are consistent with the genomic analysis and FS14 showed resistance against multiple antibiotics, which might contribute to its antagonistic effect against other microorganisms.

**Table 2:** Identification of potential antibiotic-resistant genes in *Serratia* spp.

	No. of CDSs annotated									
	FS14^a^	WW4	Db11^a^	FGI94^a^	568^b^	AS13^c^	S13^c^	Rx13^c^	ATCC 27592^d^	RB-25^e^
acriflavin	1	3	3	1	4	3	3	4	1	0
acriflavine	1	0	0	1	0	0	0	0	0	0
amikacin	1	1	0	1	0	0	0	0	0	0
aminoglycoside	2	1	0	2	2	1	2	1	1	1
bacitracin	1	1	1	1	1	1	1	1	1	1
cephalosproin	1	1	0	1	0	0	0	0	0	0
chloramphenicol	0	0	0	1	1	2	1	1	3	1
deoxycholate	1	2	1	2	3	2	2	3	2	1
dibekacin	0	0	1	0	0	0	0	0	0	0
enoxacin	1	1	0	1	1	0	0	0	1	1
fluoroquinolone	1	1	0	1	1	1	1	1	1	1
fosfomycin	0	0	0	1	0	1	0	0	0	0
fosmidomycin	2	2	2	2	2	2	2	2	2	2
kasugamycin	1	1	1	1	1	1	1	1	1	1
macrolide	3	0	4	2	2	3	2	1	2	1
na_antimicrobials	0	0	0	0	0	0	0	0	0	1
penicillin	2	2	2	4	2	2	2	2	3	2
streptogramin	0	0	0	0	0	0	0	0	1	0
streptomycin	1	1	1	1	1	1	1	1	1	0
teicoplanin	1	1	1	1	1	1	1	1	1	1
tetracycline	1	1	1	1	1	1	1	1	1	1
trimethoprim	1	1	0	1	1	1	0	0	0	1
vancomycin	0	0	0	0	0	1	1	1	0	0
Total	22	20	18	26	24	24	21	21	22	16

^a^: *S*. *marcescens*;

^b^: *S*. *proteamaculans*;

^c^: *S*. *plymuthica*;

^d^: *S*.*liquefaciens*;

^e^: *S*. *fonticola*.

**Table 3:** The antibiotic sensitivity of *S*. *marcescens* FS14.

Antibiotic	Sensitivity
azithromycin	I
acetyl spiramycin	R
amikacin	I
amoxicillin	R
ampicillin	R
cefradine	R
chlorampenicol	S
erythromycin	I
gentamicin	S
kanamycin	S
levofloxacin	S
lincomycin	R
neomycin	I
penicillin	R
polymyxin	I
rifampicin	R
spectinomycin	S
streptomycin	R
tetracycline	R
vancomycin	R

R: Resistant; S: Sensitive; I: Intermediate.

### The Type VI secretion system

**Fig 5:**
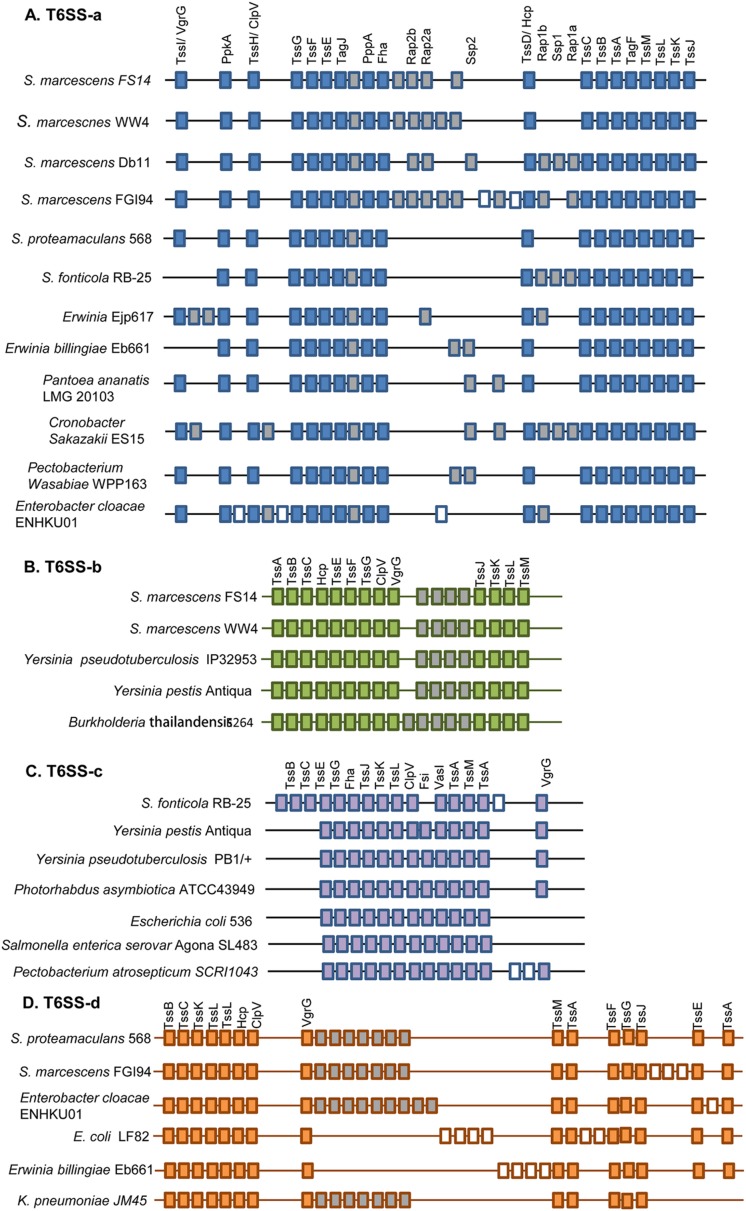
Genetic organizations of T6SS in *Serratia* and other bacteria. (A), (B), (C) and (D) show the genetic organizations of the 11 T6SS clusters found in *Serratia*, which have different organizations and separate into four families (T6SS-a, T6SS-b, T6SS-c and T6SS-d). T6SS-a was aligned across *S*. *proteamaculans* 568, *S*. *fonticola* RB-25, *S*. *marcescens* Db11, FGI 94, FS14 and WW4 genomes at the corresponding loci. T6SS-b was found in *S*. *marcescens* FS14 and WW4. T6SS-c was only found in *S*. *fonticola* RB-25. T6SS-d was found in *S*. *proteamaculans* 568 and *S*. *marcescens* FGI 94. Each T6SS cluster contains conserved T6SS core component genes. Solid blue/green/purple/orange color boxes indicate the conserved T6SS core genes; gray boxes represent variable conserved genes and white boxes are unique genes. Details of the genetic organization of T6SS clusters in *Serratia* are listed in S4 Table.

Type VI secretion system (T6SS) is widely distributed amongst the Gram-negative bacteria. It is used to deliver antagonistic effectors to either adjacent bacteria or target eukaryotic cells [10]. T6SSs are encoded by variable gene clusters consisting of 13 core essential components (TssA-M), which form the apparatus that resembles an inverted bacteriophage puncturing device [55]. Two distinct T6SS clusters of 24-kb and 28-kb were identified in the FS14 genome, both found on a putative genomic island (GI03, S1 Fig, Fig 5). Mobile DNA elements (transposases, IS elements) are found between the two T6SS clusters, which indicates that the two T6SSs are likely to have been acquired by horizontal gene transfer or internal chromosomal rearrangements. No T6SSs were identified in *S*. *liquefaciens* ATCC 27592 and *S*. *plymuthica* strains, but the other *Serratia* evaluated also contain two T6SSs, with the exception of *S*. *marcescens* Db11, which has only one T6SS (S4 Table).

Synteny analysis between the 11 T6SSs of the *Serratia* spp. showed that the T6SSs in *Serratia* separate into four families termed as T6SS-a, b, c and d (Fig 5). Only T6SS-a of the four evolutionarily distinct *Serratia* T6SSs is conserved among them. T6SS-a in strain Db10 (Db11) was previously shown to target bacterial competitors with antagonistic effectors, but is not required for virulence in eukaryotes [52]. T6SS-b in FS14 and WW4 shows synteny and homology with the T6SS of animal or soil-associated bacteria, such as *Burkholderia thailandensis* E264, which has five T6SSs including both anti-eukaryotic and antibacterial T6SSs [12]. T6SS-c in the *S*. *fonticola* RB25 genome is related to T6SSs of *Yersinia pestis* Antiqua, *Photorhabdus asymbiotica* ATCC43949 *etc*. in terms of composition, synteny and homology (Fig 5, data not shown). *Serratia* T6SS-d was found in *S*. *marcescens* FGI94 and *S*. *proteamaculans* 568.

Besides the gene variation in the core components, the accessory elements of T6SS clusters are highly variable; for example, T6SS-a of *Serratia* contains multiple accessory proteins, such as PpkA, PppA and Fha. Since the bacteria carrying T6SS gene clusters are found in diverse environments and the function of T6SS is highly versatile [10, 56, 57], these accessory proteins might be involved in regulation or might confer additional functions to the system [57]. Homologs of PpkA, PppA and Fha in T6SS-a was formerly characterized to play important roles in activation of T6SS at transcriptional or post-translational levels [58, 59]; however, the three regulatory components are absent from the other three T6SS families identified in *Serratia* (Fig 5, S4 Table). Moreover, some of the CDSs found within the T6SS cluster were reported as secretory effector proteins or self-resistance (‘immunity’) proteins, *e*.*g*. the Ssp proteins which are novel toxins recently identified in *S*. *marcescens* Db10 [60]. So it is speculated that other genes assigned with unknown functions in T6SS clusters are likely novel effectors or immunity proteins, whose roles remain to be experimentally verified.

It has been estimated that about one third of bacteria with a T6SS have multiple systems [55]. Multiple T6SSs in one organism, *e*.*g*. *B*. *thailandensis*, *P*. *aeruginosa*, may confer the species the ability to target multiple organisms [12, 61, 62, 63], allowing the secreting species to overcome rival bacteria and successfully colonize polymicrobial niches. The presence of T6SSs in FS14 may contribute to its antagonistic effect against microbial competitors to survive in polymicrobial habitats, but this hypothesis still requires further experimental elucidation.

### Prodigiosin and other antimicrobial compounds

**Fig 6:**
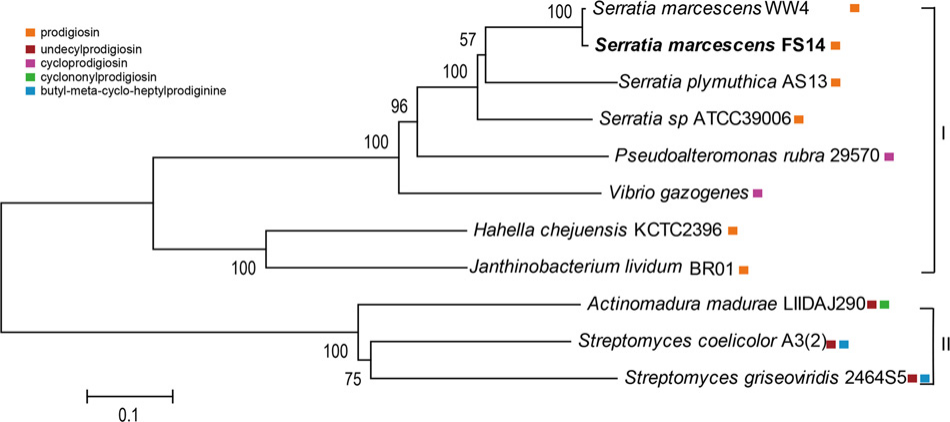
Phylogenetic tree of prodigiosin biosynthesis genes from all prodiginine-producing bacteria. The Maximum Likelihood Tree with 100 bootstrap replicates was constructed based on Poisson correction model [35] with concatenated amino acid sequences of 8 proteins, PigA(RedW), PigC(RedH), PigF(RedI), PigG(RedO), PigH(RedN), PigI(RedM), PigK(RedY) and PigM(RedV) that are involved in prodiginine-biosynthesis and commonly found among 11 prodiginine-producing bacteria. Adjacent colored squares represent different kinds of prodiginines produced by the bacteria: prodigiosin (orange), undecylprodigiosin (red), cycloprodigiosin (purple), cyclononylprodigiosin (green), and butyl-meta-cyclo-heptylprodiginine (blue).

The ability of microorganisms to produce diverse antimicrobial compounds including antibiotics, bacteriocins and other secondary metabolites plays an important role in their antagonistic activities against other microbes [64, 65]. Prodiginines are a family of red-pigmented tripyrrole antibiotics produced by bacteria such as pigmented *Serratia*, *Actinobacteria* and some marine bacteria. A putative prodiginine gene cluster containing 14 CDSs was found in *S*. *marcescens* FS14, WW4 and *S*. *plymuthica* AS13, but the cluster was absent in the other *Serratia* genomes. Phylogenetic analysis with concatenated amino acid sequences of 8 proteins commonly found in the prodiginine-producing bacteria formed two main clades (Clade I and Clade II) (Fig 6). Clade I includes *Serratia* spp. and marine bacteria which produce prodigiosin or cycloprodigiosin, while Clade II includes *Actinobacteria* strains producing other kinds of prodiginines. Orthologs of FS14 were grouped under the clade of other prodigiosin-producing bacteria, and the FS14 *pig* cluster showed high amino acid sequence identities (~75%, S5 Table) with those in *Serratia* sp. ATCC 39006, which has been used as a model for the study of prodigiosin biosynthesis [66].

**Fig 7:**
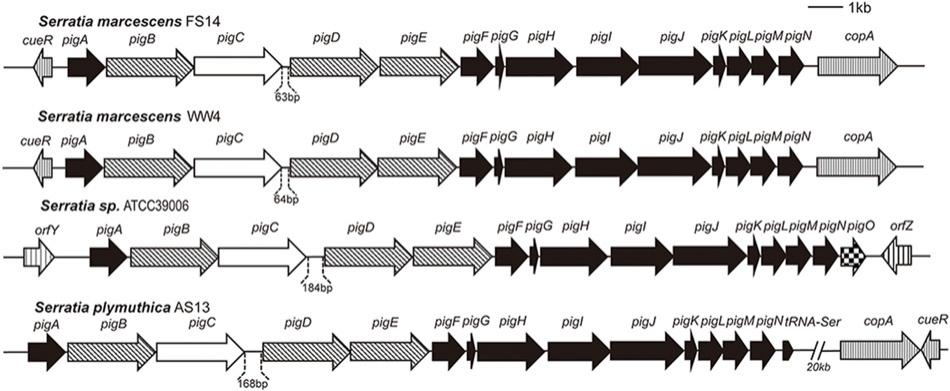
Synteny of the prodigiosin biosynthesis *pig* clusters among pigmented *Serratia* spp.. Genes are symbolized by arrows. The white arrows denote condensing enzymes; black arrows represent genes that encode proteins required for the biosynthesis of the monopyrroles; genes encoding proteins required for the biosynthesis of 4-methoxy-2,2-bipyrrole-5-carboxyaldehyde are in thin diagonally striped arrows; checkered arrows are used to highlight the additional *pigO* gene only found in ATCC39006; vertical striped arrows denote other flanking genes.

As shown in Fig 7, the synteny among the *pig* clusters of the *S*. *marcescens* FS14, WW4, *S*. *plymuthica* AS13 and ATCC3066 is conserved, except for a *pigO* present downstream of the cluster in ATCC39006. The *pig* cluster of *S*. *marcescens* FS14 is flanked by *cueR* and *copA*, genes involved in metal (copper)-dependent regulation and efflux (S5 Table, Fig 7). This observation is congruent with earlier studies [67] that the *pig* cluster was inserted between *cueR* and *copA* in the common ancestor of pigmented *S*. *marcescens*. In *S*. *plymuthica* AS13, the *cueR* and *copA* genes were directly adjacent to each other, but localized 20Kb downstream of the *pig* cluster. Moreover, a tRNA-Ser element was found directly adjacent to *pigN* in *S*. *plymuthica* AS13, hinting at the occurrence of a potential genomic rearrangement.

Many antibiotics produced by soil or plant associated bacteria have been reported to suppress diseases caused by bacterial pathogens [68]. The production of the red-pigmented prodigiosin in *S*. *marcescens* FS14 might contribute to its antagonistic effect against other bacteria. In addition, studies have shown that prodigiosin-producing isolates of *S*. *marcescens* are rarely found as human pathogens [7]; however, since *S*. *marcescens* FS14 shows a close relationship to a clinical strain and possesses multiple antibiotic resistant genes and T6SSs, further tests on its potential toxicity for humans including possible genetic modifications of the pathogenicity-related genomic elements should be carried out before further study and application.

The competition for limiting natural resources through producing bacteriocins to inhibit the growth of related organisms is also an antagonistic potential of bacteria [69, 70]. Within the ten *Serratia* genomes, 11 clusters were found to be involved in the biosynthesis of bacteriocin, colicin, entericidin, microcin and pyocin and one of the 11clusters is conserved across the genus (Table 4). FS14 has five bacteriocin gene clusters and one microcin H47 cluster (L085_22830–22835) of them is only found in the FS14 genome. Some of the bacteriocin operons also encode immunity proteins (*e*.*g*. L085_18570) as the cognate bacteriocins which may protect the producing bacterium from the inhibition by its own bacteriocin [69].

**Table 4:** Genes involved in potential bacterial competitions in *Serratia*.

Pan-genome position	Description	Representing Loci	*S*. *marcescens*				*S*. *proteamaculans*	*S*. *plymuthica*			*S*. *liquefaciens*	*S*. *fonticola*
			FS14	WW4	Db11	FGI 94	568	AS13	S13	Rx13	ATCC 27592	RB-25
**Bacteriocin**												
451–452	Entericidin	L085_02315–02320	+	+	+	+	+	+	+	-	+	-
2317–2319	Microcin C	L085_11940–11950	+	+	+	+	+	+	+	+	+	+
3308–3309	Microcin H47	L085_17075–17080	+	+	-	-	-	-	-	+	-	-
3599–3600	S-type pyocin	L085_18570–18585	+	+	+	-	+	-	+	+	-	+
4429–4430	Microcin H47	L085_22830–22835	+	-	-	-	-	-	-	-	-	-
6487–6491	S-type pyocin	SOD_c04120–04170	-	+	-	-	+	-	-	+	-	-
7063–7064	Microcin H47	SOD_c38320–38330	-	-	-	-	-	-	-	+	-	-
12127–12133	bacteriocin	SMDB11_1570–1575	-	-	+	-	-	-	-	-	-	-
12365–12366	S-type pyocin	SMDB11_4214–4215	-	-	+	-	-	-	-	-	-	-
**Chitinase**												
996	Chitinase A	L085_05105	+	+	+	-	+	+	+	+	+	+
2080	Chitinase B	L085_10730	+	+	+	-	+	+	+	+	+	-
2922	Chitinase A	L085_15120	+	+	+	-	+	+	+	+	+	+
4364	Chitinase C	L085_22490	+	+	+	+	+	+	+	+	+	+
5302	Chitinase C	SerAS13_2254	-	-	-	-	-	+	-	+	-	-

### Chitinases

Other resources subjected to antagonistic activities include lytic enzymes such as chitinases. *S*. *marcescens* is known as one of the most efficient producers of chitinases, which degrade chitin, the main component of fungal cell walls and exoskeletons of insects [71, 72]. The chitinolytic activities of bacteria are mainly attributed to 3 essential enzymes: the processive chitinases chitinase A (ChiA), chitinase B (ChiB) essential for full degradation of chitin, and endo-acting chitinase C (ChiC) [73]. Five putative chitinases are found across *Serratia* genomes (Table 4) including two ChiC, and one of the ChiC is conserved across *Serratia*. An additional ChiC is found in the *S*. *plymuthica* AS13, S13 and *S*. *fonticola* RB-25 genomes. Two of the chitinases show high identities with the well-characterized ChiA in *S*. *marcescens* which is responsible for chitin hydrolysis and was demonstrated to control several important fungal phytopathogens [74, 75]. ChiB is not present in *S*. *fonticola* RB-25, suggesting that the bacteria may not degrade chitin efficiently or that a novel ChiB functioning gene may exist. Both ChiA and ChiB are absent in *S*. *marcescens* FGI 94, implicating that it may have lost the ability to degrade chitin when it adopted a commensal life with fungus. FS14 possesses 4 genes involved in the secretion of chitinases and they may play a critical role in its suppression of fungi necessary for its competitive lifestyle in diseased plants.

FS14 possesses a set of genes involved in the secretion of chitinases and they are expected to play an important role in the inhibition of mycelial extension, which is in agreement with the observations from our co-culture assays of FS14 and fungi. In fact, the culture filtrate of some *S*. *plymuthica* and *S*. *marcescens* has been used as a biocontrol agent against fungi because of their secretion of chitinases [76, 77].

## Conclusions

In a nutshell, the complete genome of *S*. *marcescens* FS14 is 5.25Mb with a genomic GC content of 59.46%. The comparison of ten *Serratia* genomes has identified the universal core genes of *Serratia* and unique genes to each strain. *S*. *marcescens* FS14 possesses a unique Type I RM system, whereas FGI 94 has acquired a type III secretion system and *S*. *fonticola* RB-25 was found to have homologues of tellurium resistance genes. The presence of prodigiosin, bacteriocins, multi-drug resistant proteins and chitinases in *Serratia* suggests its antagonistic potential. The identification of two T6SS clusters offers further evidence that FS14 competes with other microbes, thus enhancing the survival of FS14 in various habitats. Compared to other *Serratia*, FS14 possesses both a prodigiosin cluster and two T6SSs. The antagonistic effect assays demonstrated that *S*. *marcescens* FS14 can suppress the growth of the vital plant pathogenic bacterium *Ralstonia solanacearum* and fungi *Fusarium oxysporum* and *Sclerotinia sclerotiorum in vitro*.

## Supporting Information

S1 Fig Characteristics of *Serratia marcescens* FS14 genome. From outer to inner layer: (1) nucleotide positions in kilobases (kb) (black); (2) COG database-annotated CDSs (light red); (3) ACLAME database-annotated potential horizontal transferring genes (orange: from plasmids, blue: from prophage and black: from virus); (4) ARDB-annotated potential drug resistant genes (green); (5) prodigiosin biosynthetic gene cluster (red), genes related to biosynthesis of chitinases (black), siderophores (blue); (6) tRNA region (purple), rRNA (red); (7) Predicted genomic islands (yellow); (8) GC density.
(PDF)

S2 Fig Genomic alignment of *S*. *marcescens*. Progressive Mauve [33] alignment of *S*. *marcescens* FS14, FGI 94, WW4 and Db11 genome sequences with default parameters. Each same color block represents a locally collinear block (LCB) (*i*.*e*. homologous region shared by genomes without any rearrangements). Rearrangement of genomic regions was observed in the four genomes, in term of collinearity and their localization on the negative or positive strand (indicated by their genomic position below or above the black horizontal center line in the Mauve alignment, respectively).
(PNG)

S3 Fig Phylogenetic analysis of T3SS of *Serratia*. Maximum Likelihood Tree based on amino acid sequences of the conserved T3SS ATPase associated with *S*. *marcescens* FGI 94 constructed by 44-representive orthologs from each species using MEGA5. 7 different families of T3SS were identified. T3SS in *S*. *marcescens* FGI 94 was found in the Hrp1 family, which mainly composed of plant pathogens. The ATPase of the flagellum of *E*. *coli* was used as an outgroup. Bootstrap values are shown as percentages of 100 replicates, numbers at nodes represent bootstrap values, and only bootstrap values of >50 are shown.
(TIF)

S4 Fig Comparison of COG-annotated genes between *Serratia* strains. COG-annotated genes of *S*. *marcescens* FS14 were compared to 9 other *Serratia* genomes: *S*. *marcescens* WW4 (CP003959), *S*. *marcescens* Db11 (HG326223), *S*. *marcescens* FGI 94 (CP003942), *S*. *proteamaculans* 568 (CP000826), *S*. *plymuthica* 4Rx13 (CP006250), *S*. *plymuthica* S13 (CP006566), *S*. *plymuthica AS13* (CP002775), *S*. *liquefaciens* ATCC27592 (CP006252), and *S*. *fonticola* RB-25 (CP007044).
(PDF)

S1 Table Pan-genome analysis of *Serratia* spp.. (XLSX)

S2 Table Predicted prophage regions among *Serratia*. (XLSX)

S3 Table COG functional annotation of FS14 and class distribution between *Serratia* spp.. (XLSX)

S4 Table List of genes associated with Type VI Secretion Systems in *Serratia*. (XLSX)

S5 Table *pig* cluster in FS14 and comparison with other prodiginine-producing bacteria. (XLSX)
