# Supplementary figures and images for: Comparative Genome Analyses of Serratia marcescens FS14 Reveals Its High Antagonistic Potential
Source: PLoS One. 2015 Apr 9;10(4):e0123061. doi: 10.1371/journal.pone.0123061 (PMC4391916; doi:10.1371/journal.pone.0123061)

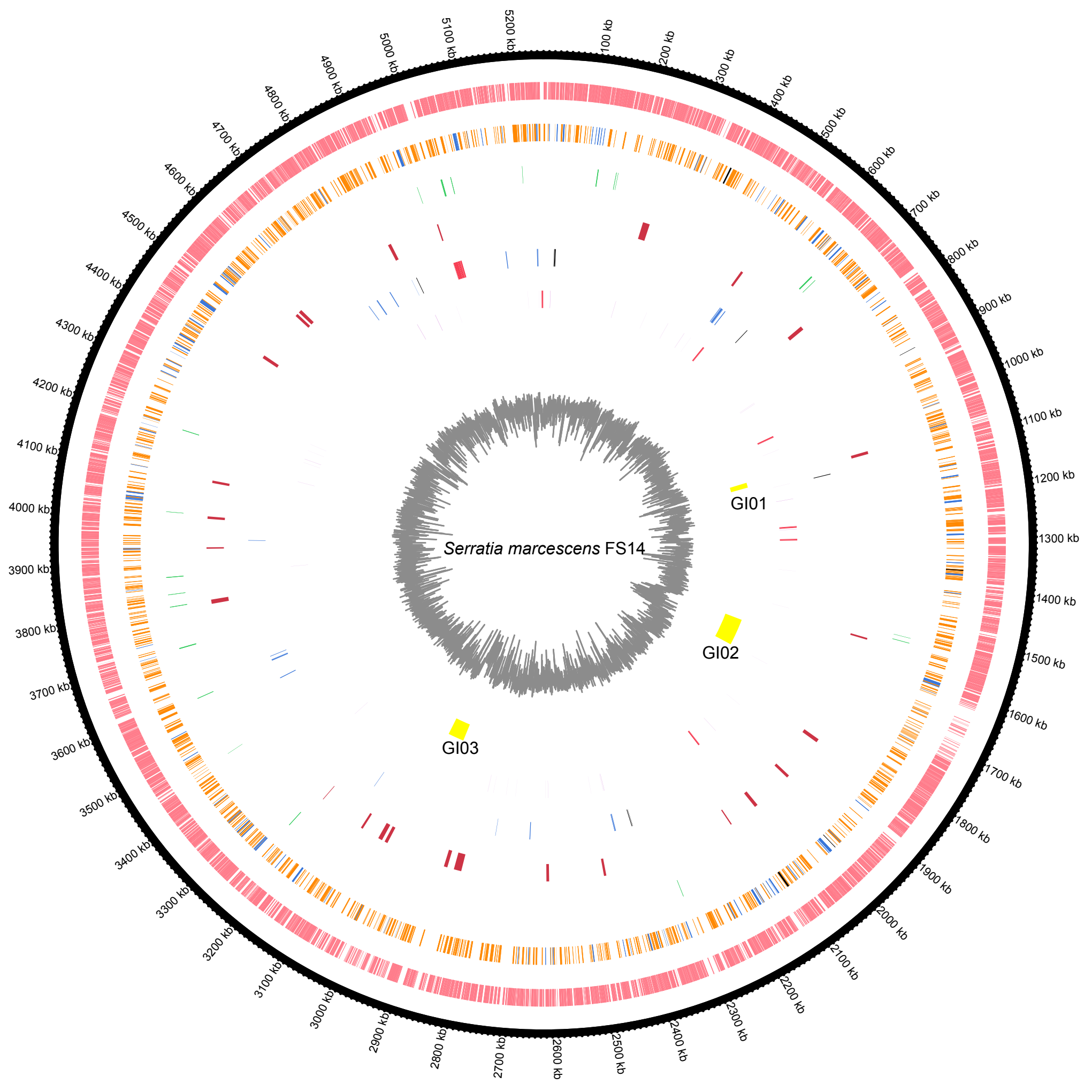

Supplement: S1 Fig — From outer to inner layer: (1) nucleotide positions in kilobases (kb) (black); (2) COG database-annotated CDSs (light red); (3) ACLAME database-annotated potential horizontal transferring genes (orange: from plasmids, blue: from prophage and black: from virus); (4) ARDB-annotated potential drug resistant genes (green); (5) prodigiosin biosynthetic gene cluster (red), genes related to biosynthesis of chitinases (black), siderophores (blue); (6) tRNA region (purple), rRNA (red); (7) Predicted genomic islands (yellow); (8) GC density. (PDF) [file pone.0123061.s001.pdf]

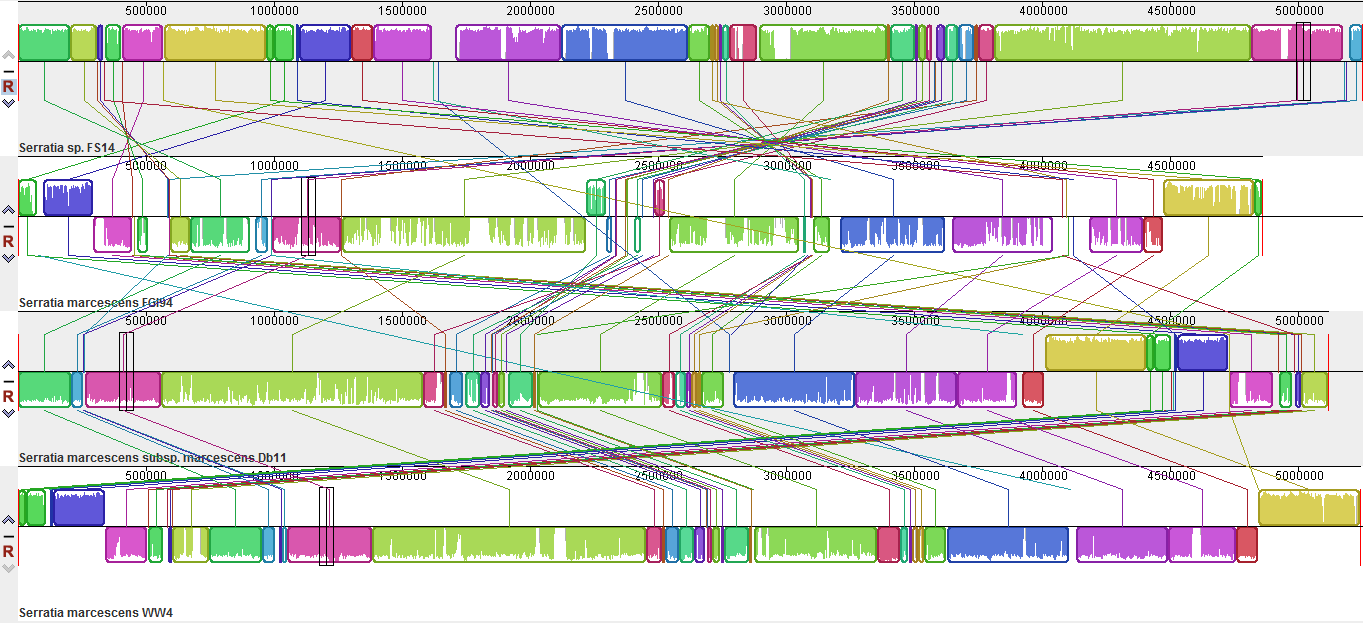

Supplement: S2 Fig — Progressive Mauve [33] alignment of S. marcescens FS14, FGI 94, WW4 and Db11 genome sequences with default parameters. Each same color block represents a locally collinear block (LCB) (i.e. homologous region shared by genomes without any rearrangements). Rearrangement of genomic regions was observed in the four genomes, in term of collinearity and their localization on the negative or positive strand (indicated by their genomic position below or above the black horizontal center line in the Mauve alignment, respectively). (PNG) [file pone.0123061.s002.png]

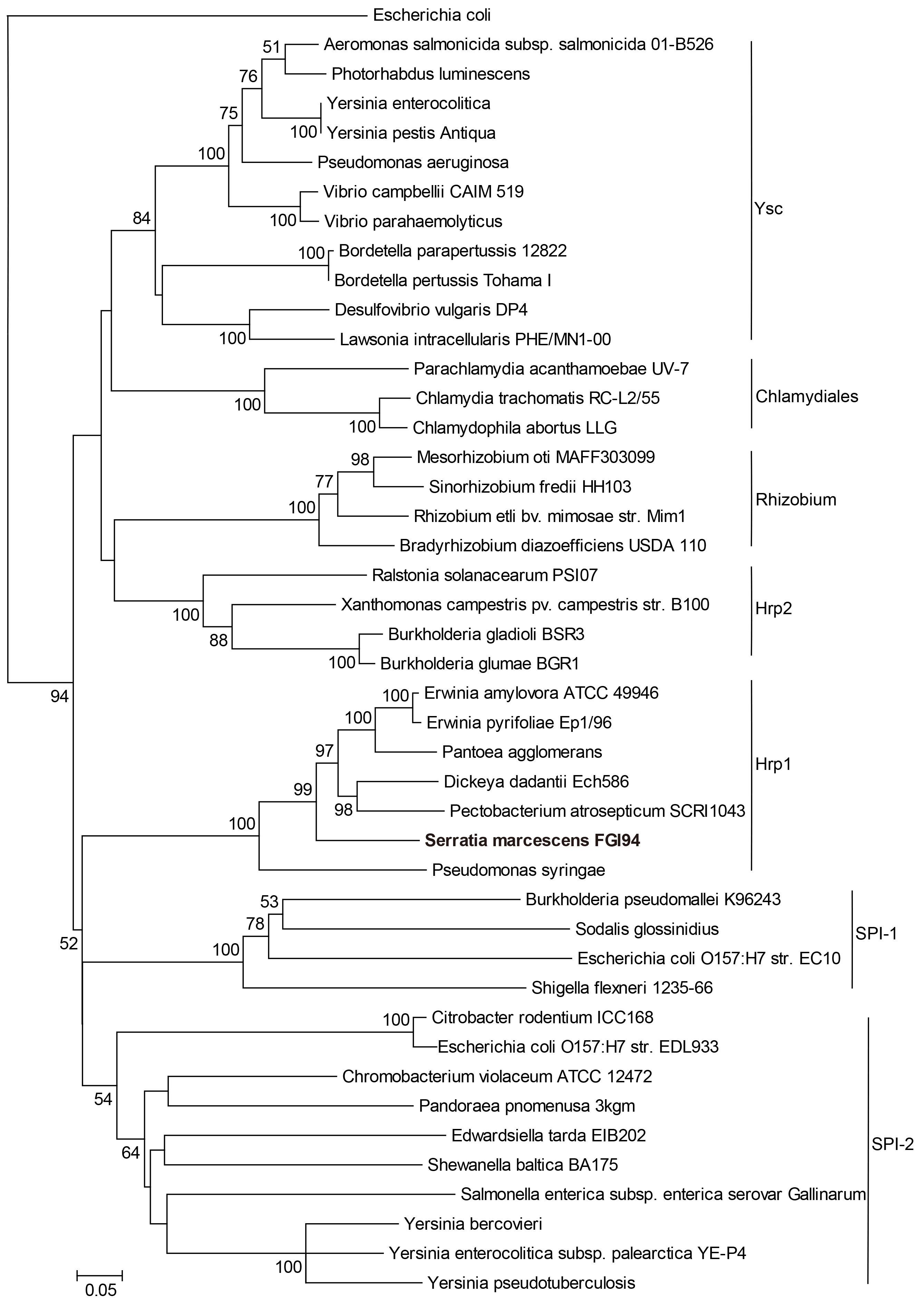

Supplement: S3 Fig — Maximum Likelihood Tree based on amino acid sequences of the conserved T3SS ATPase associated with S. marcescens FGI 94 constructed by 44-representive orthologs from each species using MEGA5. 7 different families of T3SS were identified. T3SS in S. marcescens FGI 94 was found in the Hrp1 family, which mainly composed of plant pathogens. The ATPase of the flagellum of E. coli was used as an outgroup. Bootstrap values are shown as percentages of 100 replicates, numbers at nodes represent bootstrap values, and only bootstrap values of >50 are shown. (TIF) [file pone.0123061.s003.tif]

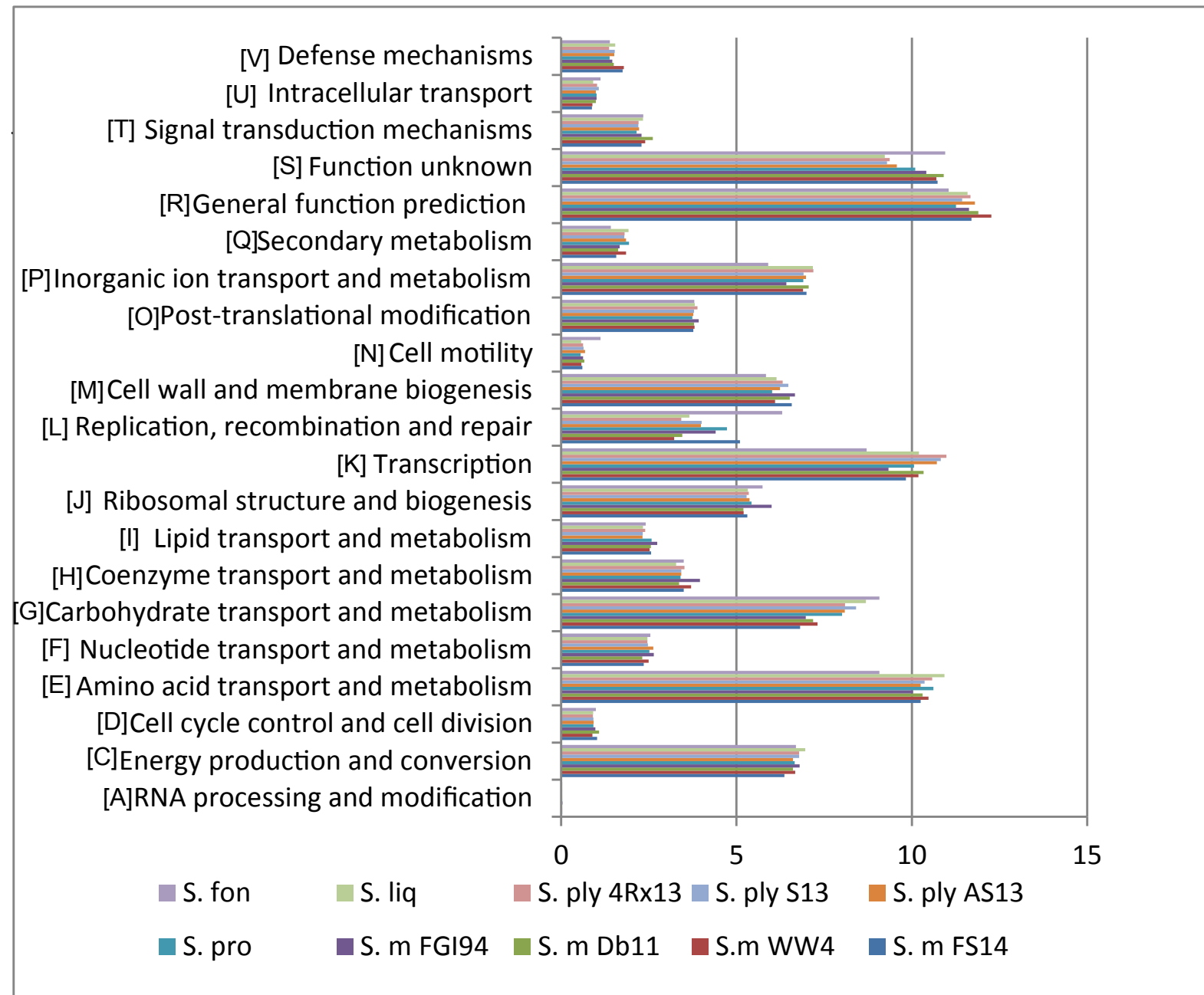

Supplement: S4 Fig — COG-annotated genes of S. marcescens FS14 were compared to 9 other Serratia genomes: S. marcescens WW4 (CP003959), S. marcescens Db11 (HG326223), S. marcescens FGI 94 (CP003942), S. proteamaculans 568 (CP000826), S. plymuthica 4Rx13 (CP006250), S. plymuthica S13 (CP006566), S. plymuthica AS13 (CP002775), S. liquefaciens ATCC27592 (CP006252), and S. fonticola RB-25 (CP007044). (PDF) [file pone.0123061.s004.pdf]
